# Cannabis use during first episode psychosis in Tunisia

**DOI:** 10.1192/j.eurpsy.2023.1592

**Published:** 2023-07-19

**Authors:** E. Bergaoui, R. Lansari, O. Chehaider, M. Moalla, A. Larnaout, W. Melki

**Affiliations:** Psychiatry D; 2 Razi hospital, Manouba, Tunisia

## Abstract

**Introduction:**

Cannabis use is frequent among patients with psychotic disorders. However, the relationship between cannabis consumption and transition to psychosis has not been fully elucidated.

**Objectives:**

The aim of this study was to assess the prevalence of cannabis use in first episode psychosis and its correlation with transistion to psychosis and severity of symptoms.

**Methods:**

A cross-sectional study was conducted at the psychiatric department D of Razi hospital including 50 patients hospitalized for first episode psychosis. The evaluation focused on sociodemographic and clinical characteristics of the patients. We used the cannabis abuse screening test (CAST) and positive and negative syndrome scale (PANSS).

**Results:**

The sex ratio of our patients was 4 men per 1 woman. The mean age was 25.6±6.16 years. Two-thirds of the patients had secondary education (n=24). Half of them had no occupation (n=17). Twenty-five patients (71%) had no psychiatric history. The total PANSS score showed a mean of 58.29±12.90 with extremes between 35 and 91. About 60% of the patients used cannabis with high addiction risk in 81% of cases. The mean duration of cannabis use was 7,04 years, 3 times a week. Cannabis use was correlated to the gender. However, no correlation was found between cannabis use and duration of untreated psychosis niether the negative or positive symptoms.

**Conclusions:**

Although cannabis use is knownto accelerate transition to psychosis, it does not affect the severity of symptoms. Further work is necessary to identify the factors that underlie individual vulnerability to cannabinoid-related psychosis and to elucidate the biological mechanisms underlying this risk.

**Disclosure of Interest:**

None Declared

